# Novel agents and regimens for hematological malignancies: recent updates from 2020 ASH annual meeting

**DOI:** 10.1186/s13045-021-01077-3

**Published:** 2021-04-21

**Authors:** Jing-Zhou Hou, Jing Christine Ye, Jeffrey J. Pu, Hongtao Liu, Wei Ding, Hong Zheng, Delong Liu

**Affiliations:** 1 Hillman Cancer Center, University of Pittsburgh Medical Center (UPMC), 5115 Centre Ave., Fl 4, Pittsburgh, PA 15232 USA; 2 Department of Internal Medicine, Hematology/Oncology Division, University of Michigan, Rogel Cancer Center, 1500 E Medical Center Dr, Ann Arbor, MI 48109 USA; 3 Department of Medicine, University of Arizona NCI Designated Comprehensive Cancer Center, Tucson, AZ USA; 4 Department of Medicine, Section of Hematology/Oncology, The University of Chicago Medical Center, 5841 S. Maryland, MC 2115, Chicago, IL 60637-1470 USA; 5 Division of Hematology, Mayo Clinic, Rochester, MN 55905 USA; 6 Penn State Hershey Cancer Institute, Penn State University College of Medicine, Hershey, PA 17033 USA; 7 Department of Medicine, New York Medical College and Westchester Medical Center, Valhalla, NY 10595 USA; 8 Chinese American Hematologist and Oncologist Network (CAHON), 555 East Wells Street, Suite 1100, Milwaukee, WI 53202 USA

**Keywords:** CAR-T, Antibody–drug conjugate, LOXO-305, Asciminib, BiTE, BCMA

## Abstract

Antibodies and chimeric antigen receptor-engineered T cells (CAR-T) are increasingly used for cancer immunotherapy. Small molecule inhibitors targeting cellular oncoproteins and enzymes such as BCR-ABL, JAK2, Bruton tyrosine kinase, FLT3, BCL-2, IDH1, IDH2, are biomarker-driven chemotherapy-free agents approved for several major hematological malignancies. LOXO-305, asciminib, “off-the-shelf” universal CAR-T cells and BCMA-directed immunotherapeutics as well as data from clinical trials on many novel agents and regimens were updated at the 2020 American Society of Hematology (ASH) Annual Meeting. Major developments and updates for the therapy of hematological malignancies were delineated at the recent Winter Symposium and New York Oncology Forum from the Chinese American Hematologist and Oncologist Network (CAHON.org). This study summarized the latest updates on novel agents and regimens for hematological malignancies from the 2020 ASH annual meeting.

## Background

Monoclonal antibodies (mAbs), bispecific T cell engagers (BiTEs) and antibody–drug conjugates (ADC) as well as immunotoxins are increasingly used for cancer immunotherapy [1–4]. Cellular immunotherapeutic agents, including the chimeric antigen receptor-engineered T cell (CAR-T) therapy and NK cell therapy, are undergoing extensive clinical investigation and development [5–14]. Small molecule inhibitors targeting cellular oncoproteins and enzymes such as BCR-ABL, JAK2, Bruton tyrosine kinase (BTK), FLT3, BCL-2, IDHs, have fundamentally transformed the landscape of cancer therapy from cytotoxic chemotherapy to biomarker-driven precision therapy [15, 16]. Combination chemotherapy (chemo) regimens have been challenged by chemo-free targeted therapies in several major hematological malignancies. Many novel agents and regimens were reported, with the latest clinical trial data updated at the 2020 American Society of Hematology (ASH) Annual Meeting. Major therapeutic developments and updates for hematological malignancies reported at ASH 2020 meeting were discussed at the recent Winter Symposium and New York Oncology Forum from the Chinese American Hematologist and Oncologist Network (CAHON.org) and summarized in this review.

### Update of non-Hodgkin lymphoma (NHL) therapy from ASH 2020 annual meeting

#### Newly diagnosed diffuse large B cell lymphoma (DLBCL)

R-CHOP (rituximab, cyclophosphamide, adriamycin, vincristine, prednisone) has been the standard frontline treatment for patients with advanced DLBCL since 2002. Many novel agents and dosing strategies appeared promising in early phase clinical trials; however, in phase 3 trials, they all failed to improve the overall survival (OS) or/and progression-free survival (PFS), including the addition of lenalidomide (R2-CHOP) or ibrutinib (IR-CHOP) [17, 18]. Nivolumab, an anti-PD-1 immune check-point inhibitor (ICI) as a single agent, was shown to have a 10% overall response rate (ORR) for relapsed or refractory DLBCL (rrDLBCL) [19].

Avelumab (Av), an anti-PDL1 mAb, was combined with R-CHOP (AvR-CHOP) as frontline therapy for patients (pts) with stage II-IV DLBCL [20]. This trial with a unique design contained three phases: prime phase with Avelumab and Rituximab q14 days for 2 cycles; R-CHOPx6 cycles; and Av maintenance phase with Avelumab single agent q14 days for 6 cycles. Twenty-eight patients at age 18 or older were enrolled. The complete metabolic response was 21% after AvR priming and 89% after 6 cycles of R-CHOP. At one year, the event-free survival (EFS) was 89% and the overall survival (OS) was 96%. The major severe adverse events (AEs) were neutropenia, febrile neutropenia and infusion reaction.

For patients with high-risk DLBCL such as double-hit (DH) or triple-hit (TH) mutations, CAR-T cell therapy was tested as frontline therapy in ZUMA-12 trial [21]. Patients with IPI score ≥ 3 (72%) or DH/TH (53%) were treated with R-CHOP like regimens for 2 cycles. Patients with interim-positive PET (Deauville value > 4) received single Axicabtagene ciloleucel (Axi-cel) infusion at 2 million cells/kg after standard fludarabine and cyclophosphamide (FC) conditioning. The ORR was 85% with 74% in complete response (CR). At a median 9.5-month follow-up, the median duration of response (DOR), progression-free survival (PFS) and OS had not been reached. The major grade 3 and 4 AEs were cytokine release syndrome (CRS) (9%), neurotoxicity (NT) (25%) and neutropenia (44%).

#### Relapsed or refractory DLBCL (rrDLBCL)

Tafasitamab, a humanized Fc-enhanced anti-CD19 mAb in combination with lenalidomide, was approved by US FDA in 2020 for patients with rrDLBCL after data from the phase 2 study (L-MIND) became available [22]. The median age of the patients was 72 (range 62–76), and the median line of therapy was 2 (range 1–4). At a median follow-up of 13.4 months, 34 of 80 patients (43%) achieved CR and 14 of 80 (18%) had a partial response (PR). This study was updated at the ASH 2020 meeting with a longer median follow-up of 22.7 months. The CR rate was 40.0% (*n* = 32/80). Median DOR was 34.6 months; median PFS was 12.1 months; and median OS was 31.6 months. Subgroup analysis showed that 86.4% patients with CR at 13.4 months remained in CR at 34.6 months, and OS was 90.6% at 24 months [23].

#### CD20-CD3 BiTEs (Table 1)

**Table 1:** CD20-CD3 bispecific T cell engager for relapsed or refractory diffuse large B cell lymphoma

Agent	Odronextamab	Mosunetuzumab	Epcoritamab	Glofitamab
Targets	CD20, CD3	CD20, CD3	CD20, CD3	CD20, CD3
IG	Human IgG4	Human IgG1	Human IgG1	Human IgG
Administration	i.v	s.c	i.v	i.v
Patients (*n*)	35	22	18	28
Median prior therapy	3 (1–11)	4 (1–8)	NA	3 (1–12)
Prior CAR T	69%	22%	NA	NA
ORR	44%	60%	67%	50%
CR	38%	20%	33%	29%
PFS	NR	NR	NR	NR
OS	NR	NR	NR	NR
CRS, any grade	63%	21%	58%	57.9%
≥ 3	5%	0	0	3.8%
NT, any grade	0	22%	9%	0
≥ 3	0	0	3%	0
Reference	[24]	[25]	[26]	[27]
	ASH,2020, #400	ASH,2020, #401	ASH,2020, #403	ASH,2020, #404

Odronextamab (REGN1979) is a hinge-stabilized, fully humanized IgG4-based CD20-CD3 bispecific antibody [24]. Bannerji et al. reported that 78 pts with rrDLBCL were treated with odronextamab at doses ranging from 0.03 to 320 mg. Out of 35 patients treated within the efficacy dose range 80-320 mg, 11 patients had no prior history of CAR-T cell therapy, and 24 (69%) patients had prior history of CAR-T cell therapy. The ORR and CR were 55% for patients without prior CAR-T therapy, and 33% and 21% for patients with prior CAR-T therapy, respectively. The duration of CR was 83% at 21 months for patients without CAR-T therapy and 100% at 20 months for patients with prior CAR-T therapy. The combined ORR and CR were 44% and 38% (Table 1), respectively. The median PFS and OS have not been reached yet. The CRS rate of any grade was 63%; grade 3 and 4 CRS rates were 5%. No neurotoxicity (NT) or tumor lysis syndrome (TLS) was observed.

Mosunetuzumab (Mosun) is another humanized IgG1 CD20-CD3 BiTE. Mosun had been shown to have promising efficacy and tolerability in an ongoing phase 1/1b trial. Schuster et al. reported that the objective response rate (ORR) was 37.1%, with a CR rate of 19.4% in 124 patients with aggressive NHL [10]. CRs appeared to be durable, with 17 of 24 patients remaining in CR for up to 16 months after stopping treatment. The major AEs were CRS (28.9%), neurologic toxicity (43.7%). To minimize the risk of CRS and neurotoxicity, subcutaneous administration of Mosun was tested in 23 patients with relapsed or refractory B-NHL [25]. The median number of prior systemic therapy was 4 (range 1–8), and 5 (22%) patients had prior CAR-T cell therapy. Among efficacy evaluable patients with aggressive NHL, the ORR and CR were 60% (9/15) and 20% (3/15), respectively. For patients with indolent NHL, the ORR and CR were 86% (6/7) and 29% (2/7), respectively. The major AEs were CRS (35%), headache (22%, all grade 1) and injection site reaction (22%, all grade 1). It appears that SC Mosun did not reduce the risk of CRS.

Epcoritamab (GENE3013, DuoBody CD20-CD3) is a fully humanized IgG1 CD20-CD3 BiTE. It has been shown to have promising efficacy and safety profile in an ongoing phase I/II trial. Hutchings et al. reported the result from updated dose escalation cohorts [26]. Out of 18 patients with rrDLBCL treated at a dose of 12 mg or higher, 6 (33.3%) patients had CR and 6 (33.3%) patients had PR. At the recommended phase 2 dose of 48 mg or higher, 2 (28.6%) patients had CR, and 5 (71.4%) patients had PR. The CR appeared durable, and 100% of patients who achieved CR remained in CR at the median follow-up of 12 months. The major AEs were pyrexia (70%), local injection reaction (48%) and fatigue (45%). The CRS rate was 58% at grade 1/2, none at grade 3/4. The neurotoxicity was transient and limited (6% grade 1, 3% grade 3).

Glofitamab is a fully humanized CD20-CD3 BiTE with bivalent binding to CD20 and monovalent binding to CD3 [27]. In a dose escalation study of patients with rrNHL, the median number of prior systemic therapy was 3 (range 1–12) and 28 (73.7%) patients had aggressive NHL. After a median follow-up of 2.8 months, 24 patients with aggressive NHL had an ORR of 50% and complete metabolic remission (CMR) of 29.2%. For the 8 patients with indolent NHL, the ORR was 100% and CMR was 75%. The major AEs were CRS (57.9%), pyrexia (31.6), neutropenia (28.9%) and thrombocytopenia (28.9%).

#### Dual-targeted CAR-T cell therapy (Table 2)

**Table 2:** Dual- versus single-targeted CAR-T cell therapy for relapsed or refractory large cell lymphoma

	CD19/22	CD19/CD20	Axi-Cel	Tisa-Cel	Liso-Cel
	(Alexander)		(Zuma-1)	(Juliet)	(Transcend)
Phase	1	1	2	2	2
Patients (*n*)	33	12	111	93	256
Target	CD19, CD22	CD19, CD20	CD19	CD19	CD19
Co-stim	OX40, 41BB	41BB	CD28	41BB	41BB
CR	52%	42%	52%	40%	53%
Median PFS (month)	NA	NA	5.9	3	6.8
Median OS (month)	NA	NA	NR	12	21.1
CRS, any grade	33%	42%	93%	58%	42%
≥ 3	NA	0	13%	22%	2%
Neurotoxicity any grade	9%	8%	64%	21%	30%
≥ 3	0	0	28%	13%	10%
References	[33]	[34]	[30]	[8]	[5]
	ASH 2020, #600	ASH 2020, #404	NEJM, 2017	NEJM, 2019	Lancet, 2020

CAR-T cell therapy targeting CD19 has offered durable remission in 40–50% of patients with relapsed or refractory aggressive lymphoma [5, 8, 28]. Four CD19-targeted CAR-T cell therapeutics have been approved by US FDA for high-risk aggressive lymphomas, including axicabtagene ciloleucel (Axi-cel) in October 2017, *tisagenlecleucel* (Tisa-cel) in May 2018, brexucabtagene autoleucel in July 2020 and lisocabtagene maraleucel (Liso-cel) in February 2021 [5, 8, 9, 29, 30]. Antigen escape or lack of adequate antigen expression contributes to the failure of CAR-T therapy [31]. Targeting two antigens simultaneously may overcome antigen escape in B cell malignancies. CD22 and CD20, the other two lineage specific markers in B cell development, are attractive targets [32]. Two early-phase clinical trials are highlighted here (Table 2).

In the AUTO3 study, the CD19/22 dual-targeted CAR-T cells were combined with pembrolizumab administrated on day 1 prior to infusion of CAR-T cells on day 0 for patients with rrDLBCL [33]. Out of 33 patients, 9 patients had double-hit and 3 patients had triple-hit mutations. The major AEs were neutropenia (73%), thrombocytopenia (64%), anemia (61%), CRS (33% grade 1 and 2, no grade 3 or 4) and pyrexia (30%). NT occurred in 3 (9%) cases in the setting of disease progression. The ORR and CMR were 69% and 52%. Fourteen of 15 (93%) patients who achieved CR remained in CR at a median of 3-month follow-up. A cohort of the patients was treated in the outpatient setting.

In a phase I trial of MB-CART2019.1, a CD19/20 dual-targeting CAR-T cell product was tested at two dose levels in 12 patients, including 11 patients with aggressive rrNHL and 1 patient with rrMCL [34]. No grade 3 or higher CRS or NT was reported. The ORR was 75% and CR was 42%. Those patients who attained CR remained in CR with a maximum of 330-day follow-up or at the data cutoff. Of note, MB-CART2019.1 was produced by lentiviral transduction in a closed automated system, which makes it practically possible that the CAR-T cells can be produced onsite at each institution.

Previous studies of Axi-Cel, Tisa-Cel, and Liso-Cel have shown that the CR rate ranged from 40% to 53% for relapsed or refractory aggressive NHL (Table 2). Only 65%-76% of patients who had achieved CR remained in CR during long-term follow-up. Will dual-targeted CAR-T cells improve the outcome of relapsed or refractory aggressive lymphoma? Large phase 2 studies are ongoing to address the question.

#### Follicular lymphoma (FL) and marginal zone lymphoma (MZL)

Frontline therapy for indolent NHL such as FL and MZL includes options of wait/watch, single-agent anti-CD20 mAb for low tumor burden and chemoimmunotherapy for high tumor burden.

ZUMA-5 studied Axi-Cel in 124 patients with rrFL and 22 patients with rrMZL [35]. The median line of prior systemic therapy was 3 (range 1–10), 64% patients had 3 lines or more therapy, 55% patients had progression within 2 years after last chemoimmunotherapy. With a median follow-up of 17.5 months, the ORR was 92% and CR rate was 76%. In rrFL patients, the ORR was 94% with a CR rate of 80%. In rrMZL, the ORR was 85% with a CR rate 60%. The median DOR, PFS and OS were not reached yet. At 12 months, the estimated DOR was 72%, PFS 74% and OS 93%. More than 99% of patients had AE of any grade. The most common grade ≥ 3 AEs were neutropenia (33%) and anemia (23%). The grade ≥ 3 CRS was 7%, and grade ≥ 3 NT was 19%. One patient died from multiorgan failure in the context of CRS; 1 patient died from aortic dissection at day 399; and 1 patient died from coccidiomycosis at day 327; both were deemed to be not related to Axi-Cel by the study investigators. Out of the patients who relapsed after the first Axi-Cel infusion, 11 patients were treated with second autologous Axi-Cel. Ten patients achieved CR and 1 attained PR. With a median follow-up of 2.3 months, the median DOR was not yet reached, and 82% patients had ongoing response at the time of data cutoff. Based on the responses and duration of responses from the ZUMA-5 trial, US FDA granted accelerated approval of Axi-Cel for rrFL after two lines of therapy on March 5, 2021.

In a phase 2 open label multicenter trial (MAGNOLIA), Opat et al. reported data on zanubrutinib, a second-generation irreversible BTK inhibitor, in 68 patients with relapsed or refractory marginal zone lymphoma (rrMZL) [36]. The median age of patients was 70 years (range 37–95), median lines of prior system therapies were 2 (range 1–6), and 35% patients had refractory disease to last therapy. At a median follow-up of 6.8 months, the ORR was 60% with CR rate 15%. CR was not observed in patients with splenic MZL. The median DOR and median PFS have not been reached. The major treatment-emergent AEs (TEAE) of any grade were diarrhea (19.1%), bruising (17.6%), constipation (13.2%), pyrexia (10.3%), upper respiratory tract infection (10.3%) and nausea (10.3%). The most common grade ≥ 3 AE was neutropenia (7.3%). Other treatment-related serious AEs included atrial flutter, pneumonia (1 patient each). No major hemorrhage, serious opportunistic infection or tumor lysis syndrome was reported.

#### Relapsed or refractory mantle cell lymphoma (rrMCL)

Chemoimmunotherapy remains the frontline therapy for most patients with MCL. Aggressive induction therapy followed by autologous stem cell transplant and maintenance therapy provides long-term duration of remission in young and fit patients. Patients with relapsed or refractory MCL have options of BTK inhibitors, venetoclax and autologous CD19-CAR-T cell therapy.

Ibrutinib, acalabrutinib, zanubrutinib and orelabrutinib are irreversible BTK inhibitors [37–40]. They share similar on-target activity but differ in off-target side effects (Table 3). LOXO-305 is a reversible, non-covalently bound BTK inhibitor at an allosteric binding site. In a multicenter phase I/II trial, Wang et al. reported the results of single-agent LOXO-305 in 186 pts with B-cell malignancies (94 CLL/SLL, 38 MCL, 19 DLBCL, 17 WM, 6 FL, 5 MZL and 7 other lymphomas [41]). Out of 38 MCL patients, 92% patients received a prior BTKi; 87% patients received immunochemotherapy or/and BTKi; 26% patients received SCT/CAR-T. Among 35 patients treated across all dose levels, the ORR was 51% with 25.7% CR. Among patients treated at the RP2D (200 mg QD), the ORR was 65% with 35% CR. Three of 7 patients with prior SCT, 1 of 2 with prior CAR-T had responses to LOXO-305.

**Table 3:** BTK inhibitors for relapsed or refractory mantle cell lymphoma

Parameter	Ibrutinib	Acalabrutinib	Zanubrutinib	Orelabrutinib	LOXO-305
Patients (*n*)	111	124	86	97 (106)	38
Age (years)	68	68	60.5	NA	69
Prior SCT	11%	18%	3.5%	NA	25%
Prior BTKi	NA	NA	NA	NA	93%
ORR	67%	80%	84%	87.9%	52%
CR	23%	40%	67.5%	27.4%	25%
DOR (months)	17.5	25.7	19.5	NR	NR
Median PFS (months)	13.0	NR	22.1	NR	NR
Median OS %, months	47%, 24	87%, 12	84%, 12	88.7%, 12	NR
New A Fib	11%	0%	0%	0%	< 1%
Neutropenia, severe	17%	11%	19.8%	NA	NA
Pneumonia, severe	6%	6%	9.3%	NA	NA
Bleeding, severe	6%	2.4%	2.3%	0	< 1%
Reference	[40]	[39]	[38]	[37]	[41]

A fib, atrial fibrillation; BTKi, Bruton tyrosine kinase inhibitor; NA, not applicable; NR, not reached

#### CAR-T cell therapy and novel agents for rrMCL

In July 2020, the FDA approved brexucabtagene autoleucel (KTE-X19, Tecartus) for treatment of adult patients with rrMCL based on a pivotal phase 2 trial [9] (Table 4). In this trial, 31% of 68 MCL patients had blastoid or pleomorphic characteristics, 17% patients had TP53 mutation, and 43% patients had autologous SCT. At a median follow-up of 12.3 months, the ORR was 93% and CR rate was 67% among 60 efficacy- evaluable patients. The 1-year estimated PFS was 61%, and OS was 83%. Twenty-four percent of the patients died on study, including 21% died from progressive disease, and 3% grade 5 AEs due to organizing pneumonia from the conditioning chemotherapy and one case of staphylococcus bacteremia. Wang et al. updated this trial with a longer median follow-up of 17.5 months, and the ORR was 92% with CR rate of 67% [42]. The median DOR, PFS and OS have not been reached. The 15-month estimated PFS was 59.2%, and OS was 76%. The major grade ≥ 3 AEs were neutropenia (85%), thrombocytopenia (53%) and anemia (53%). Grade ≥ 3 CRS rate was 15%, and grade ≥ 3 NT was 31%. No new grade 5 events were noted during the additional follow-up period.

**Table 4:** Selected CAR-T cell trials in relapsed or refractory mantle cell lymphoma

	ZUMA-2	ZUMA-2	Transcend
	(KTE-X19)	Update	(Liso-Cel)
Patient	*n* = 68		*n* = 34
Prior SCT	43%		40%
Prior BTKi	100%		87.5%
TP53 mutation	17%		22%
ORR	93%	92%	84%
CR	67%	67%	59%
Median DOR	NR	NR	NR
PFS	61%	59.2%	NR
OS	83%	67%	NR
CRS, any	91%	NA	50%
≥ 3	15%	15%	0%
Neurotoxicity, any	63%	NA	28%
≥ 3	31%	31%	9%
Neutropenia ≥ 3	85%	85%	34%
Anemia ≥ 3	50%	53%	31%
Thrombocytopenia ≥ 3	51%	53%	34%
TLS ≥ 3	NA	NA	3%
Grade 5 AE	3%	0	0
Reference	[9]	[42]	[43]
	NEJM, 2020	ASH, 2020	ASH 2020, #118

In the Transcend NHL-001 trial update [43], 32 patients with rrMCL received the treatment of Liso-cel. Thirty-seven percent of the lymphoma had blastoid morphology, 22% had TP53 mutation, and 34% had complex cytogenetics; 87.5% patients had prior BTKi, 72% patients had refractory disease to the last treatment, and the median line of prior systemic therapy was 3 (1–7). At a median follow-up of 10.9 months, the ORR was 84% with CR rate 59%. The median DOR, PFS and OS were not reached at the data cutoff point. The major grade ≥ 3 AEs were neutropenia (34%), thrombocytopenia (31%), anemia (34%). Grade 1 and 2 CRS rate was 50%, and no grade ≥ 3 CRS was reported. The grade 1 and 2 NT were 19%, grade 3 NT was 9%, no grade 4 or 5 NT. Other grade 3 AEs occurred in 2 patients: 1 subject had TLS and high tumor burden, and 1 subject had cryptococcal meningoencephalitis.

Receptor tyrosine kinase-like orphan receptor 1 (ROR1), an oncofetal protein, is highly expressed on the surfaces of various cancers but not on normal differentiated cells [44]. It is involved in many key processes including cancer cell proliferation, survival and metastasis. Therapeutic approaches targeting ROR1 include mAb and CAR-T cells.

VLS-101 is a ROR1-targeting humanized antibody–drug conjugate (ADC) with a cleavable linker to the anti-microtubule cytotoxin, monomethyl auristatin E (MMAE) [45]. In a first-in-human phase 1 trial, 32 patients were enrolled with different hematological malignancies. Among 15 patients with rrMCL, 100% patients had received BTKi, including 87% patients who progressed on BTKi. The ORR was 47% with CR rate 13%. The major grade ≥ 3 AEs were neutropenia (53%), neurotoxicity (13%) and diarrhea (9%). Drug infusion reaction, vomiting, skin rash, liver or renal laboratory abnormality or QT prolongation was not observed.

### Update of MM therapy from ASH 2020 annual meeting

The highlight of ASH 2020 in therapeutic advances for multiple myeloma (MM) is undoubtedly BCMA-targeted immunotherapy, led by BITEs, CAR-T/NKs cells and ADC, followed by new CD38 monoclonal antibodies and the newer generation of immunomodulatory drugs (IMIDs) (Table 5).

**Table 5:** Selected studies for MM therapy from 2020 ASH annual meeting

Abstract #	Authors (reference)	Study agents	Phase	NCT No
551	Facon et al. [49]	Ixazomib/Len/Dex	III	01850524
549	Kaufman et al. [51]	Dara/Len/Vel/Dex	II	02874742
130	Alsina et al. [57]	BB21217	I	03274219
134	Costello et al. [59]	P-BCMA-101	I/II	03288493
129	Mailankody et al. [62]	ALLO-715	I	04093596
293	Rodriguez et al. [66]	TNB-383B	I	03933735
290	Chari et al. [67]	JNJ-7564	I	03399799
292	Cohen et al. [68]	BFCR4350A	I	03275103
179	Kumar et al. [70]	MEDI2228	I	03489525
724	Van De Donk et al. [72]	Iberdomide with Dara/Dex or Vel/Dex	I/II	02773030

Len, lenalidomide; Dex, dexamethasone; Dara, daratumumab; Vel, velcade (bortezomib)

### Newly diagnosed MM (NDMM), transplant ineligible: daratumumab and ixazomib in the frontline setting

Daratumumab (Dara), the first FDA-approved CD38 mAb, has clearly demonstrated its therapeutic benefit, and set the new standard for future myeloma drug approval (failed IMIDs, proteosome inhibitors and CD38 mAb) [46]. Dara in combination with lenalidomide and dexamethasone (Rd) (MAIA trial), or with bortezomib, melphalan, prednisone (VMP) (ALCYONE trial), was recently approved by the FDA as the frontline therapy for transplant ineligible NDMM [47, 48]. In the latest update of the phase 3 TOURMALINE-MM2 study, the triple oral combination of ixazomib plus Rd (IRd) improved the median PFS by 13.5 months (35.3 vs 21.8 mo, *p* = 0.073, HR = 0.83, 95% CI 0.676–1.018) over Rd [49]. Although this PFS difference was not statistically significant, this all-oral triplet regimen with acceptable toxicity profile may offer a reasonable option during the COVID19 pandemic.

### NDMM, transplant eligible: daratumumab-based quadruplet regimen in the frontline setting

In the transplant-eligible patient population, phase 2 GRIFFIN study tested addition of daratumumab to RVd (Dara-RVd) [50]. The efficacy and safety results were updated after a longer median follow-up of 26.7 months [51]. The CR and MRD negativity rates were improved in the study arm (Dara-RVd followed by transplant and 12mo maintenance Dara-R) vs control arm (RVd for induction followed by transplant and R maintenance). CR or better was 81.8% in the study arm vs 60.8% in the control arm, and the deep MRD remission (10^–6^ threshold) was 26.9% in study arm vs 12.6% in control arm.

The phase 3 FORTE trial tested the role of autologous stem cell transplant (ASCT) in 315 newly diagnosed patients who were randomized to receive either carfilzomib, lenalidomide and dexamethasone (KRd) plus ASCT or KRd alone for 12 months (KRd-12mo). At a median follow-up of 45 months, the median PFS was not yet reached for KRd-ASCT vs 57 months for KRd-12mo (HR 0.64, *p* = 0.023) [52]. This FORTE trial confirmed that KRd-ASCT significantly improved PFS when compared with KRd-12mo.

### Relapsed refractory multiple myeloma (RRMM)

B-cell maturation antigen (BCMA) is a cell surface protein widely expressed in MM cells and is currently the most common target studied in RRMM [14]. BiTE, CAR-T/NK and ADC agents targeting BCMA have epitomized novel immunotherapy in RRMM. These BCMA-targeted agents bring therapeutic benefits to RRMM patients with advantages and disadvantages. The optimal combination and sequencing of these agents call for further studies in the future [53].

#### CAR-T cellular therapy in RRMM

Safety and efficacy data on BCMA-targeted CAR-T cells were updated at the 2020 ASH meeting for bb2121 (idecabtagene vicleucel; Ide-cel) [7, 54], CARTITUDE-1 (Cilta-cel) [55] and Lummicar-2 (CT053) [56] (Table 6). FDA recently approved Abecma [ide-cel] as the first BCMA-directed CAR-T immunotherapy for patients with RRMM based on the pivotal KarMMa trial [54].

**Table 6:** Recent updates on CAR-T cell therapy for multiple myeloma

	Ide-Cel (bb2121) (KarMMA)	bb21217 (CRB-402)	Cilta-Cel (CARTITUDE-1)	CT053 (LUMMICAR-2)	Orva-Cel (EVOLVE)	FasT (GC012F)	P-BCMA-101 (PRIME)	Universal ALLO-715
Phase	2	1	1b/2	1b/2	1/2	1	1/2	1
Patients (*n*)	128	46	97	14	44	16	43	15
Target	BCMA	BCMA with PI3Ki bb007	2-epitope BCMA	BCMA	BCMA	Dual BCMA-CD19	BCMA	BCMA
ORR/CR/MRD	73%/33%/26%	55%/18%/NA	95%/56%(sCR)/94.2%	100%/29%/92%	91%/39%/84%	93.8%/NA/56.3%	57%/NA/NA	33%/NA/NA
Median PFS (month)	8.8mo	NA	Not reached	NA	NA	NA	NA	NA
Median OS (month)	19.4mo	NA	Not reached	NA	NA	NA	NA	NA
CRS, any grade	84%	67%	94.8%	86%	NA	87.5%	17%	24%
CRS, > = grade3	5%	4% (one grade 5)	4.1%	0%	2%	12.5%	2%	0%
Neurotoxicity, any grade	18%	22%	20.6%	7%	NA	0%	2%	0%
Neurotoxicity, > = grade3	3%	7%	10.3%	0%	4%	0%	0%	0%
Reference	[54] NEJM 2021	[57] ASH 2020 #653	[55] ASH 2020 #177	[56] ASH 2020 #133	[58] ASCO 2020 #8504	[61] ASH 2020 #178	[59] ASH 2020 #134	[62] ASH 2020 #129

NA, not available; ORR, overall response rate; CR, complete response; MRD, minimal residual disease; PFS, progression-free survival; OS, overall survival; CRS, cytokine release syndrome

It is worth mentioning that bb21217 has the same CAR molecule as that in bb2121 (Ide-cel), but a PI3K inhibitor motif bb007 was added to the CAR construct so that during ex vivo expansion, memory-like T cells can be enriched to reduce the proportion of highly differentiated or senescent T cells. Initial efficacy results with bb21217 were encouraging, and 48% of patients treated across target dose levels of 150–450 million CAR-T cells achieved ≥ VGPR. The presence of memory T cell markers and the absence of differentiated/senescent T cell markers in the product correlated positively with peak expansion and DOR [57]. Other modified BCMA CAR-Ts, including orvacabtagene autoleucel (EVOLVE trial) [58], P-BCMA-101 (manufactured using a novel transposon-based system called piggyBac and designed to increase efficacy while minimizing toxicity) [59]; C-CAR088 (manufactured in a serum-free, automated and closed system, vein to vein 16 days) [60]; and GC012F (BCMA-CD19 dual target FasT CAR-T)[61], all updated their trial data.

Universal CAR-T cell therapy offers off-the-shelf benefit and is particularly attractive for patients with rapidly progressive RRMM. ALLO-715 contains a disrupted TCR alpha constant gene to reduce the risk of graft-versus-host disease (GvHD) and a disrupted CD52 gene to permit the use of ALLO-647, an anti-CD52 mAb, for selective and prolonged host lymphodepletion. This study had 19 pts enrolled and 15 received ALLO-715 at 3 dose levels (DLs): 3 pts at DL1 (3 FCA and 0 CA), 7 pts at DL2 (4 FCA and 3 CA) and 5 pts at DL3 (3 FCA and 2 CA). No DLTs, neurotoxicity or graft-vs-host disease (GVHD) from ALLO-715 had been reported as of the data cutoff. CRS was reported in 4 pts (24%), all grade 1 or 2 resolved without tocilizumab or corticosteroids. All DL3 pts experienced at least a very good PR (VGPR), observed at day 14 and achieved measurable residual disease (MRD)-negative status by local MRD testing [62].

#### BiTEs targeting BCMA, GPRC5D and FcRH5

BCMA-targeted BiTEs, AMG701 [63], JNJ 7957 (Teclistamab) [64] and REGN5458 [65] were reported/updated at this ASH meeting (Table 7). TNB-383B is a fully humanized triple chain BCMA-CD3 BiTE, with 2 BCMA domains allowing for cell surface BCMA binding, silenced IgG4 backbone to prevent nonspecific T cell activation and a unique CD3 moiety to minimize CRS. In a phase 1 study, 38 subjects were treated with TNB-383B q3w at dose levels of 0.025–40 mg. An ORR of 52% (12/23) was observed at doses of 5.4–40 mg and ORR of 80% at doses ≥ 40 mg. The CRS occurred in 45% subjects (all grade 1-2) [66].

**Table 7:** A comparison of BCMA-targeted treatment modalities in RRMM

	CAR-T/NK	BITEs	ADC
Logistics	Manufacturing time bridging therapy required Hospital infrastructure	Off the shelf	Off the shelf
Setting	Inpatient	Inpatient- > outpatient	Outpatient
Treatment	One time treatment	Mostly weekly dosing Until PD	Q3w dosing Until PD
Clinical benefit	ORR 80% Promising PFS and MRD data	ORR 60–80% PFS data N/A	ORR 30% Modest PFS with single agent
Adverse effects	CRS 70–90% (Grade > = 3: 5–10%) Neurotoxicity Lymphodepletion	CRS 35–80% (> = Grade3: 5–10%) Neurotoxicity	Keratopathy (> = Grade3: 20–25%) Thrombocytopenia
Host T cell dependency	Yes except in universal CAR-T/NK	Yes	No

RRMM, refractory relapsed multiple myeloma; ORR, overall response rate; PFS, progression-free survival; MRD, minimal residual disease; CRS, cytokine release syndrome; q3w, every 3 weeks

Talquetamab (JNJ-64407564) is a first-in-class bispecific antibody that binds to GPRC5D and CD3 to induce T cell-mediated killing of GPRC5D-expressing MM cells. In this phase 1 study, 137 pts received talquetamab: 102 by IV (0.5–180 µg/kg) and 35 by SC (5–800 µg/kg) dosing. The most common grade 3–4 AEs were lymphopenia (37%), anemia (27%) and neutropenia (25%). CRS was mostly grade 1–2 except for 5 pts with grade 3 CRS that occurred with IV dosing; only grade 1–2 CRS was seen with SC dosing. Treatment-related neurotoxicity was reported in 7 (5%) pts. ORR for IV doses of 20–180 µg/kg was 78% (14/18; 2 pending confirmation); 6/6 responded at the 60 µg/kg IV dose. ORR for SC doses of 135–405 µg/kg was 67% (8/12); 3/4 responded at the 405 µg/kg SC dose [67].

FcRH5 (Fc receptor-homolog 5) is a type I membrane protein that is expressed on B cells and plasma cells, and it is found on myeloma cells with near 100% prevalence. BFCR4350A is a BiTE that targets the most membrane-proximal domain of FcRH5 on myeloma cells and CD3 on T cells. The phase 1 trial was conducted in 51 patients. At the 3.6/20 mg dose level and above, ORR was observed in 15/29 pts (51.7%), including 3 stringent CRs, 3 CRs, 4 VGPRs and 5 PRs. Those responsive patients had history of high-risk cytogenetics (9/17); triple-class refractory disease (10/20); and prior exposure to anti-CD38 mAbs (11/22), CAR-Ts (2/3) or ADCs (2/2). CRS grade 1 occurred in 20 pts (39.2%), grade 2 in 17 patients (33.3%) and grade 3 in 1 patient (2%). No grade 4 CRS was reported [68].

#### Novel BCMA ADC in RRMM

Belantamab Mafodotin-blmf is the first BCMA-targeted ADC approved by US FDA, based on an open-label, multicenter trial, DREAMM-2, for patients with RRMM [69]. MEDI2228 is a novel BCMA ADC specifically conjugated to a DNA cross-linking pyrrolobenzodiazepine (PBD) dimer via a protease-cleavable linker. Once bound to BCMA, MEDI2228 is internalized and cleaved in the lysosomal compartment. The active PBD is released and it cross-links DNA and leads to apoptotic cell death. In a phase 1 study, 82 patients received MEDI2228 during dose escalation and expansion. In the 0.14 mg/kg cohort, ORR was 61.0%; there were 10 (24.4%) VGPR and 15 (36.6%) PR. The major AEs were photophobia (53.7%), thrombocytopenia (31.7%), skin rash (29.3%), elevated gamma-glutamyltransferase (24.4%), dry eyes (19.5%) and pleural effusion (19.5%). No keratopathy or visual acuity loss was observed in this cohort [70].

#### New IMID on the horizon in RRMM

Iberdomide (IBER, CC-220) is an oral potent novel cereblon E3 ligase modulator (CELMoD) agent [71]. It was combined with dexamethasone and daratumumab (IberDd) or bortezomib (IberVd) in a phase I/II study. In the IberDd arm, the major grade 3–4 AEs included neutropenia (50%), leukopenia (22%) and anemia (22%). In the IberVd arm, the major grade 3 and 4 AEs were neutropenia (20%) and thrombocytopenia (20%). In the IberDd cohort, 12/19 (63%) patients were dara refractory and 11 (58%) patients were quad-class refractory. The ORR was 35% (2 VGPR, 4 PR) across all dosing groups. In the IberVd cohort, 16/21 (76%) patients were PI refractory, 9 (43%) patients were bortezomib refractory, and 10 (48%) patients were quad-class refractory. The ORR was 50% (1 CR, 3 VGPR, 6 PR) [72]. How to best combine and/or sequence the newer generation of IMID remain to be addressed in future clinical trials.

### Update of MPN therapy from ASH 2020 annual meeting

Historically, therapeutic phlebotomy, hydroxyurea and interferon-alpha (IFN) were the main supportive care measures for treating myeloproliferative neoplasms (MPN). Ruxolitinib was the first drug approved by FDA to treat patients with intermediate or high-risk primary or secondary myelofibrosis (MF) and a subgroup of polycythemia vera (PV) patients who have inadequate response or intolerant to hydroxyurea [73]. Two retrospective studies presented at the 2020 ASH annual meeting investigated the impacts of ruxolitinib dose alteration on first-line treatment outcomes at the real-world settings (Table 8). Abstract 3442 summarized data from 183 patients. This study reported that 65 patients initiated the treatment in the label-recommended doses, and 75 patients initiated the treatment in the modified doses due to various medical reasons [74]. The investigators found that dose modifications occurred in nearly 1/3 of those patients, with the majority requiring a dose reduction. Abstract 2518 is a multinational medical record review on clinical outcomes of 135 patients with myelofibrosis who received ruxolitinib as the first-line treatment [75]. Among the 135 MF patients, 99 received stable doses and 36 received modified doses. The data from both studies demonstrated that many patients started with non-standard ruxolitinib dose, either higher or lower than the recommended doses, were associated with lower responses, poorer survival outcomes and higher rates of discontinuation due to disease progression. It was also shown that relatively few patients received subsequent treatment after ruxolitinib discontinuation. These data highlighted a significant unmet need for developing newer and more effective MF treatment strategies**.**

**Table 8:** Selected studies for myeloproliferative neoplasm from 2020 ASH annual meeting

Abstract #	Authors (references)	Study agents	Phase	NCT No
3442	Kish et al. [74]	Ruxolitinib	Retrospective	N/A
2518	Passamonti et.al. [75]	Ruxolitinib	Retrospective	N/A
1250	Drummond et.al. [77]	Vidaza/Ruxolitinib	1b	ISRCTN 16783472
54	Verstovsek et al. [82]	Momelotinib	Extension Study	N/A
3002	Potluri et.al. [87]	Navitoclax/Rux	III	NCT04472598
1255	Dilley et al. [86]	Navitoclax/Rux	III	NCT04468984
52	Pemmaraju et al. [88]	Navitoclax/Rux	II	N/A
483	Daltro De Oliveira et al. [90]	Interferon-Alpha	N/A	N/A

Inspired by the success of hypomethylating agents as a treatment component in managing relapse/refractory AML [76], a phase 1b Phazar trial tested the safety and efficacy of azacitidine/ruxolitinib combination for MPN patients who were either in “accelerated phase” **(**MPN-AP, 10–19% blasts) or in post-MPN AML phase (MPN-BP, ≥ 20% blasts). All those patients were ineligible to receive hematopoietic stem cell transplantation (HSCT) [77]. A modified two-stage continual reassessment method with an expansion cohort at the maximum tolerated dose (MTD), was used to establish the MTD of ruxolitinib (dose levels 0, 1, 2 and 3 = 10, 15, 20 and 25 mg twice daily, respectively) in combination with a fixed azacitidine dose of 75 mg/m^2^ subcutaneously for 7 days of a 28-day cycle. A formal response assessment would be recorded after 6 cycles. Clinical activity was evaluated over 12 months through assessment of bone marrow response after 3 and 6 treatment cycles, as well as of PFS, leukemia-free survival (LFS) and OS. There were 34 patients enrolled in this study, 20 evaluable for disease response. The data from this study demonstrated that the azacitidine/ruxolitinib combination was well tolerated in both MPN-AP and MPN-BP patients. The toxicities were comparable to the individual agent. Of the 20 evaluable patients, 10 (50%) achieved a PR or CR. The OS compares favorably with that of the historical cohorts, and clinically meaningful responses were achieved for transfusion independence. One patient was successfully bridged to HSCT.

Momelotinib (MMB) is a potent JAK1, JAK2 and ACVR1 inhibitor with clinical activity against the three hallmark features of MF [78]. Previously conducted phase 3 SIMPLIFY-1 & -2 clinical trials (S1, S2) verified the feasibility and efficacy of MMB as the first-line or second-line treatment for intermediate- or high-risk MF patients [79, 80]. The abstract 54 further evaluated the impacts of 10-year MMB exposure on those patients [81, 82]. Of the 137 pts who have been enrolled in the long-term follow-up study after participating in S1 or S2 study, 105 patients remained on MMB with therapy duration ranging up to 10 years. The data showed that in both S1 and S2, OS and LFS were similar between treatment groups (stratified HR for OS of 0.99 in S1, 0.96 in S2). Overall, 40% of patients randomized to MMB in S1 achieved a splenic response at any time during the study. For patients who achieved transfusion independence (TI) at any time during the study, the median duration of TI response was not yet reached in S1 and was > 1 year in S2. These data demonstrated MMB’s potential ability to durably address the unmet needs of patients with intermediate-/high-risk MF. Fedratinib is another FDA-approved selective JAK2 inhibitor [83]. Long-term safety data of fedratinib in patients with intermediate- or high-risk myelofibrosis (MF) from JAKARTA trial were also updated [84].

Navitoclax is a novel BCL-2 inhibitor that demonstrated cell-killing activity in MPN-derived cell lines and primary specimen ex vivo [85]. Abstracts 3002 (Transform-1) and 1255 (Ttransform-2) investigated the combination of navitoclax/ruxolitinib in treating naïve and refractory/relapsed (RR) MF, respectively [86, 87]. Interestingly, abstract 52 evaluated the efficacy of adding navitoclax to ruxolitinib in treating RRMF patients who bear high-risk mutations (HRM) (ASXL1, SRSF2, EZH2, U2AF1 and IDH1/2) in a phase 2 study [88]. In that study, a total of 34 patients continued ruxolitinib therapy while also starting on daily navitoclax at a dose of 50 mg, which was escalated to 300 mg based on the patient’s tolerability. Mutation analyses were performed at baseline, 12 weeks and 24 weeks. The data showed that clinical improvement at 24 weeks was independent of HRM mutations and number of mutated genes. Interestingly, the investigators observed a correlation between percentage change of inflammatory cytokines in serum and the change of spleen volume.

Life-long treatment represents a major burden for patients with chronic MPN. Interferon-alpha **(**IFN) appears to be the only drug that can provide long-term complete hematological remission (CHR) after discontinuation in some patients [89]. Abstract 483 presented a study that aimed to identify clinical and molecular factors associated with long-term CHR after IFN treatment discontinuation and to compare clinical outcome of patients who discontinued therapy, to patients who continued IFN treatment despite achieving a CHR [90]. A total of 381 patients on IFN were enrolled in this study (PV = 171, ET = 169 and PMF = 34). JAK2V617F was the most frequent driver mutation (78.8% of patients), while *CALR* and *MPL* were mutated in 15.5% and 2.9%, respectively. After a median follow-up of 72.4 months [range 28.4 -119.7] from IFN initiation, 131 patients were still on IFN treatment, while 250 patients had discontinued therapy. No significant difference was observed between patients who discontinued and those who continued IFN in terms of MPN subtype, initial clinical, biological or molecular characteristics. OS (HR 0.23, 95%CI [0.5; 1.14], *p* = 0.07) and EFS (HR 0.53, 95%CI [0.19; 1.45], *p* = 0.217) were not significantly different between the two groups. This study showed that IFN discontinuation represents a safe strategy for MPN patients who achieved CHR and particularly for patients with a driver VAF lower than 10% at the time of discontinuation. Importantly, those relapsed patients did not develop IFN resistance.

### Update of CML therapy from ASH 2020 annual meeting

Currently, four tyrosine kinase inhibitors (TKIs), imatinib, dasatinib, nilotinib and bosutinib, have been approved by FDA for the front-line treatment of chronic myeloid leukemia in chronic phase (CML CP) [91]. While second-generation TKIs could produce faster and deeper responses and low rate of disease progression, they failed to improve the long-term OS when compared with imatinib. Thus, the NCCN and ELN guidelines still recommend imatinib for CMP CP with low-risk disease defined by Sokal score or the latest European Treatment and Outcome Study (EUTOS) long-term survival (ELTS) score [92–94].

The approval of bosutinib for front-line CML-CP was based on early results of BFORE trial [95] (Table 9). The final five-year follow-up results of BFORE were presented at ASH 2020. At 5-year follow-up, first-line therapy with bosutinib continued to show superior efficacy than imatinib to induce earlier and deeper molecular responses. An improvement in molecular remission (MR) with bosutinib was demonstrated across Sokal risk groups, with the greatest benefit in Sokal high-risk patients. Long-term AEs were generally manageable and consistent with previously reported and known safety profiles. These results confirm the use of bosutinib as a standard of care in patients with newly diagnosed CML-CP [96]. Among the second-generation TKIs, most clinicians select one of them, depending on the toxicity profile for front-line treatment of CML-CP, assuming equal efficacies. The Japanese Adult Leukemia Study Group (JALSG) should be applauded for conducting a prospective randomized phase 3 study to compare nilotinib *vs* dasatinib in achieving MR4.5 at 18 months for newly diagnosed CMP-CP. Basically, consistent with common assumption, nilotinib and dasatinib are equally effective in achieving MR4.5 as well as in achieving complete cytogenetic remission (CCyR) and major molecular remission (MMR) in terms of both frequencies and times to achievement with similar continuity. Safety profiles from both drugs were also consistent with the known AEs [97].

**Table 9:** Selected studies for CML therapy from 2020 ASH annual meeting

Abstract #	Authors (reference)	Study	Phase	NCT
46	Brummendorf et al.[96]	Bosutinib versus imatinib	III	NCT02130557
45	Matsumura et al. [97]	Nilotinib versus dasatinib	III	#UMIN000007909
632	Cortes et al. [100]	Ponatinib	II	NCT02467270
650	Cortes et al. [103]	Asciminib	I	NCT02081378
LBA-4	Hochhaus et al. [104]	Asciminib versus bosutinib	III	NCT03106779
651	Jiang et al. [108]	HQP1351 (Olverembatinib)	II	NCT03883087 NCT03883100
652	Cortes et al. [109]	Vodobatinib	I	NCT02629692

Ponatinib is a third-generation TKI, and the first TKI to exhibit activity against CML with T315I mutation. Ponatinib was initially approved in December 2012 under the FDA’s accelerated approval program based on the phase II PACE trial with daily dose of 45 mg. One of the main concerns for ponatinib was arterial occlusive events (AOEs) that occurred in 31% of patients (26% serious) [98]. The marketing of ponatinib was suspended per the request from FDA in 2013 due to the concern for AOEs, and in 2014, ponatinib resumed marketing with label changes to narrow the indication, to provide additional warnings and precautions about the risk of blood clots and severe narrowing of blood vessels, to revise recommendations about dosage and administration of ponatinib. FDA granted ponatinib full approval for the treatment of adult patients with CML and Ph + ALL with T315I mutation or for whom no other TKI therapy is indicated in 2016 based on updated PACE results [99]. The OPTIC (NCT02467270) post-marketing study was started in 2015 to understand the optimal ponatinib dose with three starting doses (45 mg, 30 mg and 15 mg daily) in patients with CML CP. At the ASH 2020 meeting, Dr. Cortes presented the interim analysis of the OPTIC trial. With a median follow-up of 21 months, the maximum benefit/risk ratio, regardless of mutation status or number of prior TKIs, was observed in patients treated with a 45 mg starting dose, with a reduction to 15 mg upon achievement of response of ≤ 1% BCR-ABL IS (International Scale). Patients with the T315I mutation who initiated ponatinib at 45 mg experienced better response rates than those who initiated ponatinib at 30 mg or 15 mg starting doses [100]. On 12/18/2020, FDA approved the supplemental new drug application for ponatinib to treat patients with CMP CP with resistance or intolerance to at least 2 prior kinase inhibitors. There was an updated label to include an optimized, response-based dosing regimen with a daily starting dose of 45 mg and, upon achieving ≤ 1% BCR-ABL1 IS, dose reduction to 15 mg to maximize benefit over the risk.

Asciminib is an allosteric inhibitor that binds to a myristoyl site of the BCR-ABL1 protein, locking BCR-ABL1 into an inactive conformation through a mechanism distinct from the other approved TKIs. Asciminib targets both native and mutated BCR-ABL1, including the gatekeeper T315I mutant [101]. In the Phase 1, dose-escalation study, asciminib was administered once or twice daily (at doses of 10–200 mg). The median follow-up was 14 months among patients with CML CP. Asciminib produced 92% complete hematologic remission (CHR); 54% CCyR, 48% durable MMR, including 57% MMR in patients deemed to have resistance to or unacceptable side effects from ponatinib, as well as 28% durable MMR in patients with a T315I mutation. Dose-limiting toxicities included asymptomatic elevations in the lipase level and clinical pancreatitis [102].

At ASH 2020, Dr. Cortes presented the updated efficacy and safety results in patients with T315I mutation. A total of 52 patients with T315I mutation received asciminib 200 mg BID. Among evaluable patients not in MMR at baseline, 23/49 (46.9%) achieved MMR; the Kaplan–Meier-estimated rate of durable first MMR among patients who achieved MMR was 87% (95% CI, 68.4–100.0) at 96 weeks and remained unchanged until 144 weeks. By 24 weeks, 57.1% of ponatinib-naive patients and 28.6% of ponatinib-pretreated patients achieved MMR. Asciminib is a promising therapeutic option for patients with CML CP/AP with T315I mutation, including those for whom ponatinib treatment has failed [103]. In a phase III randomized trial, patients who have failed two TKIs without T315I, V299L mutation were randomized to either bosutinib or asciminib. The efficacy and safety results from ASCEMBL, a multicenter, open-label, phase 3 study of asciminib *vs* bosutinib in patients with CML CP previously treated with ≥ 2 TKIs were presented as a late-breaking abstract (LBA 4) at ASH 2020 [104]. In total, 233 patients with CML CP who had received at least 2 prior TKIs were randomized 2:1 to receive asciminib at 40 mg twice daily (*n* = 157) or bosutinib at 500 mg once daily (*n* = 76). The rate of CCyR at 24 weeks was 40.8% and 24.2% with asciminib and bosutinib, respectively, with a common risk difference of 17.3% (95% CI 3.62–31.0%). MMR rates were 25.5% with asciminib and 13.2% with bosutinib, for a difference of 12.2 percentage points (95% CI 2.19–22.3; 2-sided *p* = 0.029). MR4 and MR4.5 at 24 weeks were also higher with asciminib at 10.8% and 8.9% vs 5.3% and 1.3%, respectively. Regarding safety, all-grade and grade 3 or higher AEs were similar between the two arms (89.7% and 50.6% with asciminib vs 96.1% and 60.5%, with bosutinib). ASCEMBL data demonstrated statistically significant superior efficacies with asciminib compared with bosutinib and a favorable safety profile, supporting the use of asciminib as a new treatment option in CML CP, particularly in patients with resistance or intolerance to at least 2 TKIs.

It has been controversial or unclear about the predictive values of additional chromosome changes and cancer-related gene or other gene mutations in addition to the t(9;22)(q34;11.2). Complex variant translocations (CVT) involving one or more additional chromosome changes were identified in less than 5% of newly diagnosed CML patients. There were conflicting reports about the prognostic impact of CVT in response to TKIs and the role of front-line treatment with imatinib or second-generation TKIs in patients with CVT. The Italian Campus CML, a network of Italian physicians involved in the management of CML patients, conducted a retrospective study on the role of CVT [105]. CVT were identified in 112 (3.3%) patients from a whole population of 3,389 subjects with newly diagnosed CML CP. Ninety-eight out of 112 patients (87%) exhibited three-way translocations, with chromosome 1, 4, 6, 10, 11, 12, 14, 15 and 17 representing the most common additional partners. Four- and five-way translocations were identified in 13 and 1 patients, respectively. The subtype of CVT had an impact on response and long-term outcomes. Patients with CVT involving chromosome 1, 4, 6, 11 or 12 had a higher frequency of MMR at 12 months than patients with CVT involving chromosome 10, 14, 15 or 17 (75.8% *vs* 30.4%, respectively, *p* = 0.001), higher frequency of stable deep molecular remission (DMR) (48.7% *vs* 22.2%, respectively; *p* = 0.04), regardless of the type of front line TKI and the ELTS score. Due to its retrospective nature, this study does not allow to define which therapy is for CML harboring CVT at diagnosis, but optimal responses treated with second-generation TKIs were statistically higher than that treated with imatinib.

The Australian CML group assessed the impact of genomic events in 160/210 newly diagnosed CML CP patients enrolled on their TIDEL II clinical trial. They identified a novel class of Ph-associated events, which were associated with the formation of the Ph chromosome. Both cancer-associated mutation and Ph-associated events were found in 16% (25 out of 160) patients, and 5 patients (3% of total patients) had both events; and 45 patients (28%) had at least 1 genomic event. Both cancer-related mutations and Ph-associated events at diagnosis were associated with inferior PFS and MR and increased risk to progress to advanced phase or development of a BCR-ABL1 kinase domain mutation. Through the univariate and multivariate modeling, cancer-related gene mutations, Ph-associated events and the ELTS score were independent predictors of failure-free survival (FFS), failure to achieve MMR and MR4. Despite a proactive strategy for TKI switch and a higher imatinib starting dose, the presence of cancer-related gene mutations or Ph-associated events conferred inferior outcomes [106].

In addition, two promising third-generation TKIs were presented at ASH 2020. HQP1351 (Olverembatinib, Ascentage Pharma, China), an oral third-generation TKI with low affinity against other kinases, had demonstrated promising efficacies in the phase I trial for CML patients who have failed prior TKIs and/or acquired T315I mutation [107]. HQP1351 was granted fast-track designation by the US FDA on May 7, 2020. The results of pivotal phase II trials were presented at the ASH 2020 meeting. HQP1351 was highly efficacious in heavily TKI-pretreated patients with T315I-mutation CML CP or CML AP and was well tolerated. In 41 evaluable CML CP patients who did not have a CCyR at baseline, 31 (75.6%) achieved MCyR, including 27 (65.9%) CCyR and 4 (9.8%) partial CyR (PCyR). Total 20 out of 41 (48.8%) evaluable patients achieved MMR. In the 23 evaluable CML AP patients without MCyR at baseline, 12 (52.2%) patients achieved MCyR, including 9 (39.1%) CCyR and 3 (13.1%) PCyR. A total of 6 out of 23 (26.1%) evaluable patients achieved MMR [108]. The safety profile was similar to that observed in the phase I trial, and no new safety signal was observed.

Furthermore, vodobatinib, a novel third-generation TKI effective against wild-type and mutated BCR-ABL1 with limited off-target activity, was evaluated in a phase I multicenter dose-escalation study in CML patients who failed ≥ 3 TKIs or less (if not eligible for other approved third-generation TKIs) (NCT02629692) [109]. The activity and safety of vodobatinib were evaluated in both ponatinib-treated and ponatinib-naïve CML CP patients. As of Jul 15, 2020, 31 CML CP patients received vodobatinib at doses of 12–240 mg. At the highest dose of 240 mg, two dose-limiting toxicities were reported including grade 3 dyspnea and grade 2 non-cardiac chest pain and grade 2 shortness of breath due to fluid retention. The recommended Phase II dose was 174 mg daily. The AE profiles in both ponatinib-treated or ponatinib-naïve patients were acceptable and expected. As for efficacies, MCyR was seen in 68% CML CP patients, with deepening of molecular responses over time; MMR was achieved in 38% ponatinib-treated population. Comparable and promising efficacy was noted in both ponatinib-treated (50% CCyR) and ponatinib-naive (67% CCyR) groups, meriting further study of vodobatinib as a potential new agent for treatment of previously treated CP-CML.

In summary, these studies presented at ASH 2020 meeting will likely shape the future landscape of clinical management of CML patients, particularly those with T315I mutation and/or highly refractory cases.

### Update of CLL therapy from ASH 2020 annual meeting

ASH 2020 provided important updates for two registration trials containing venetoclax either as front-line (CLL14) or in the relapsed setting (MURANO) for chronic lymphoid leukemia (CLL) therapy [110]. Novel combination of BTK inhibitor, venetoclax with or without CD20 antibody have been further tested and appeared to be highly efficacious. MRD-based endpoint as a surrogate marker for PFS has been increasingly used in clinical trials. CAR19-T with ibrutinib appeared to be efficacious for CLL refractory to both BTK and BCL-2 inhibitors.

### Update for first-line therapy of CLL

#### CLL14 trial

After a median 52.4-month observation, the 4-year PFS rate was 74% for CLL patients randomized in the group of venetoclax and obinutuzumab (Ven-O), significantly better than 35.4% for CLL patients in the group of chlorambucil and obinutuzumab [111] (Table 10). Approximately 74% of Ven-O-treated patients reached undetectable MRD (uMRD) at the end of therapy. Among the MRD-positive patients at the end of therapy, half of them displayed a trend of MRD decrease from cycle 7 to the time point of 3-month post therapy, and another half had a trend of MRD increase. Therefore, it is proposed that the duration of venetoclax therapy may be extended in the cohort with continuous MRD decrease to achieve uMRD eventually. However, the optimal duration of venetoclax therapy remains to be determined. The clonal growth rate in Ven-O arm was slower than the one in the control arm. In addition, the CLL patients who achieved uMRD with partial remission appeared to maintain similar PFS as the ones with uMRD and complete remission [112]. These results further added the evidence to support the use of Ven-O in time-limited duration for CLL patients requiring first-line therapy.

**Table 10:** Selected studies for CLL therapy from 2020 ASH annual meeting

Abstract #	First author (reference)	Study agents	Phase	NCT	Study name
127	Al-Sawaf [111]	Venetoclax, obinutuzumab	III	NCT02242942	CLL14
1310	Al-Sawaf [112]	Venetoclax, obinutuzumab	III	NCT02242942	CLL14
123	Wierda [113]	Ibrutinib, venetoclax	II	NCT02910583	CAPTIVATE
3138	Jain [115]	Ibrutinib, venetoclax	II	NCT02756897	
2216	Davids [114]	Acalabrutinib, venetoclax, obinutuzumab	II	NCT03580928	
1307	Soumerai [116]	Zanubrutinib, venetoclax, obinutuzumab	II	NCT03824483	BoVen
125	Kater [117]	Venetoclax, rituximab	III	NCT02005471	MURANO
124	Hillmen [120]	Ibrutinib, venetoclax	II	ISCRTN13751862	CLARITY
543	Gribben [119]	Umbralisib, ublituximab	III	NCT02612311	UNITY-CLL
542	Mato [121]	Loxo-305	I/II	NCT03740529	BRUIN
545	Benjamini [122]	CD19-CART	I	NCT02772198	
126	Mato [123]	DTRM-555	I	NCT02900716	
544	Wierda [124]	Liso-cel, ibrutinib	I	NCT03331198	TRANSCEND-004
546	Siddiqi [125]	Liso-cel	I	NCT03331198	TRANSCEND-004

#### CAPTIVATE study

The combination of ibrutinib and venetoclax was tested in CAPTIVATE study for untreated CLL patients who were younger than 70 year old. Seventy-two percent of patients achieved uMRD in BM after 12 cycles of therapy [113]. These uMRD patients were further randomized at a ratio of 1:1 to receive either ibrutinib or placebo. The disease-free survivals in these two groups were similar after 16-month follow-up. These data support discontinuation of therapy at one year in uMRD patients. The patients who were MRD positive at one year were randomized at a ratio of 1:1 to receive either ibrutinib or ibrutinib and venetoclax, and the trial is ongoing for further evaluation. The overall PFS at 3 year was about 95% in the entire cohort.

Several groups presented updated results for BTK inhibitor and venetoclax, or the combination of triplet therapy with BTK inhibitor (acalabrutinib or zanubrutinib), venetoclax and obinutuzumab as the initial treatment for CLL [114–116]. Overall, these combinations were highly efficacious, and the marrow uMRD rate ranged from 50% to around 89% after one year of total therapy. After 2 to 3-year observation, overall PFS at 3 year appeared to be in the range of 95%. Multiple phase 3 studies testing the similar combinations in comparison with different standard treatments are ongoing (NCT04608318, NCT03701282, NCT03737981, etc.).

### Update for relapsed and refractory CLL

#### MURANO

The update presented for MURANO study that enrolled relapsed/refractory CLL (rrCLL) patients without prior exposure to BTK inhibitor in one of the two arms, venetoclax and rituximab (VenR) or bendamustine and rituximab, confirmed the median PFS being 53.6 months in the venetoclax-containing arm [117]. This was similar to the median PFS of 53 months for ibrutinib when tested in rrCLL patients. It was also reported that conversion from uMRD at the end of therapy with VenR to positive MRD took approximately 19 months. Additional 25 months were required to convert MRD positive to clinical progression in this subset of patients. Most patients who eventually progressed after achieving uMRD at the end of therapy exhibited high-risk features including del (17p) or complex karyotypes or unmutated IGvH. A subset of these progressive patients received VenR treatment again and had a shorter PFS about 23.6 months. Another published report showed BTK inhibitor treatment achieved over 90% ORR and was associated with a PFS of 34 months in progressive CLL after having developed resistance to venetoclax [118]. Collectively these two studies demonstrated that venetoclax and rituximab are an effective approach for relapsed CLL patients and BTK inhibitor or reuse of Ven-R in uMRD patients from the first Ven-R will be efficacious for further disease progression.

#### UNITY-CLL

This is a phase 3 study that was designed to test the efficacy of umbralisib and ublituximab (U2) in comparison with chlorambucil and obinutuzumab in therapy naïve and rrCLL patients [119]. A total of 210 patients were enrolled into each arm. After a median follow-up of 37 months, the median PFS (31.9 months) in the U2 arm was significantly better than that in the control arm (17.9 months). The PFS of U2 arm in the rrCLL cohort was 19.5 months. Classic adverse events including transaminitis, colitis and opportunistic infection that associated with PI3Kδ inhibitor occurred in 5 to 17% of enrolled patients.

#### CLARITY

Fifty-four rr CLL patients who had prior exposure to chemoimmunotherapy (CIT) (80%) or idelalisib (20%) were enrolled into CLARITY study and received the combination of ibrutinib and venetoclax [120]. The rate of uMRD was 48% and 58%, while marrow and blood were analyzed, respectively, after completing one year of therapy. The blood uMRD rate improved from 58% to over 60% when the therapy duration extended from one year to two years. However, no further improvement was detected from year 2 to year 3. In addition, more than 2 log of disease reduction within the first 2 months of venetoclax treatment associated with higher rate of uMRD at the end of one-year therapy.

#### BRUIN

LOXO-305 is a non-covalent BTK inhibitor which binds to mutant BTK with highly selective BTK-binding capacity. In a phase 1 study that enrolled more than 170 rr CLL patients, 80% of these patients were exposed to prior BTK inhibitor and 30% of them also exposed to prior BCL-2 inhibitor. The ORR was impressive (50%) in this heavily pre-treated cohort, though with relative short follow-up. These data indicated using non-covalent BTK inhibitor is a promising approach for CLL patients refractory to covalent BTK inhibitor and BCL-2 inhibitor. Importantly, side effects appeared to be mild. Bruising, rashes, arthralgia, atrial fibrillation and hypertension only occurred in 16%, 11%, 5%, 5% and 5% of patients [121].

#### Update for Richter’s transformation

In the era of novel agents, CLL transformed into aggressive lymphoma, particularly DLBCL as Richter’s transformation (RT), remains an area of unmet clinical needs. Two studies tried to address this clinical niche in ASH 2020. CAR19-T cell approach was tried in a group of 9 RT patients who were heavily pre-treated with CIT, BTK inhibitor with or without BCL-2 inhibitor in CLL phase followed by R-CHOP for RT phase. Six out of 9 patients achieved CR after CAR-T infusion, and 3 were able to proceed with allogeneic HSCT. Two CR patients progressed and were salvaged with additional chemotherapy or PI3K inhibitor [122]. The second study (DTRM-555) utilized a concept of synthetic lethality that employed the combination of inhibitors targeting three signals, BTK, mTOR and immune-modulating agents (pomalidomide) with sub-therapeutic doses. In RT cohort (*n* = 13), 40% ORR was observed and responsive patients maintained remission for more than 4 months. This combination appeared to be tolerable with cytopenia as predominant adverse events [123].

#### CAR-T update

A cohort of 19 CLL patients with prior exposure to BTK inhibitor and half also exposed to venetoclax was tested in a phase 1 trial TRANSCEND CLL 004 using liso-cel with ibrutinib [124]. Over 95% of patients had high-risk features including 75% of cohort with TP53 mutation. The efficacy was clear with 95% ORR and 63% CR. Impressively, 79% achieved uMRD in the marrow. After 10-month follow-up, 3 out of 19 progressed, the rest of responders maintained responses. Typical AEs of CAR-T therapy including 75% all grade CRS and 32% neurological events were reported. Similarly, an update for TRANSCEND 004 monotherapy with liso-cel revealed 82% ORR. After 24-month follow-up, median PFS for double-refractory CLL patients to BTK and BCL-2 inhibitors and the entire cohort was 13 and 18 months, respectively [125]. Notably, 4 out of 11 patients in double-refractory cohort developed RT. Collectively, CAR-T targeting CD19 appeared to be effective approach to treat double-refractory CLL to both BTK and BCL-2 inhibitors, particularly with the combination with ibrutinib.

### Update of AML therapy from ASH 2020 annual meeting

The landscape of treatment for acute myeloid leukemia (AML) has significantly changed in the last 3 years with several FDA-approved new agents becoming available for our patients [126–128]. Venetoclax and agents targeting specific mutations (gilteritinib for Flt3 mutation, ivosidenib/enasidenib for IDH1/2 mutations, respectively) are now widely used for AML therapy [129, 130]. However, how to integrate or sequentially apply each treatment is challenging. In addition, despite these advances, the overall prognosis of AML remains poor. Novel effective therapeutics is an urgent unmet need. Although there were no guideline-changing reports for AML in ASH 2020, several studies provide valuable insights for clinical practice applying the recently approved agents (Table 11). Importantly, there are multiple new drugs under development that are promising [128].

**Table 11:** Selected studies for AML therapy from 2020 ASH annual meeting

Abstract #	Authors (reference)	Study agents	Phase	NCT
27	Wang et al. [131]	Gilteritinib	III	02752035
333	Daver et al. [136]	Gilteritinib, Venetoclax	Ib	03625505
461	Pollyea et al. [137]	Azacitidine, Venetoclax	Sub study of III and Ib	02203773 02993523
636	Stein et al. [138]	Enasidenib	Sub study of II	03013998
330	Sallman et al. [143]	Magrolimab	Ib	03248479
331	Aldoss et al. [144]	Flotetuzumab	I	02152956
460	Ravandi et al. [145]	Vibecotamab	I	02730312
165	Abedin et al. [146]	Lintuzumab Ac225	I	03441048

#### Gilteritinib combined with azacitidine as first-line treatment for Flt3-mutated AML

The LACEWING study is a phase 3 trial applying gilteritinib to newly diagnosed AML patients ineligible for intensive induction chemotherapy. The preliminary results were reported [131]. The study design involves a safety cohort followed by a randomization between 2 arms: gilteritinib plus azacitidine vs. azacitidine alone. The safety cohort was to establish dose of gilteritinib to be used in combination with azacitidine. Among the 15 patients enrolled, five (33%) achieved CR and 10 (67%) composite CR (CRc). The median DOR was 10.4 months for the CRc responders. The combination treatment was well tolerated with no unexpected adverse effect. Dose of 120 mg daily for gilteritinib was set for the randomization cohort, which is currently ongoing. These data provide a promising option of gilteritinib plus azacitidine for newly diagnosed *Flt3*-mutated AML. It is worth noting that in the VIALE-A trial, subgroup analysis demonstrated a superior CRc in *Flt3*-mutated AML patients receiving azacitidine plus venetoclax compared with azacitidine alone (72% vs. 36%, *p* = 0.02) [132]. Based on these data, combining azacitidine with either gilteritinib or venetoclax is an appealing therapeutic option for first-line treatment of Flt3-mutated AML who are unfit for intensive chemotherapy. Whether one is superior to the other awaits further studies.

#### Gilteritinib in combination with venetoclax for relapse/refractory Flt3-mutated AML

Gilteritinib monotherapy has been FDA approved for relapse/refractory (rr) *Flt3*-mutated AML based on the data from ADMIRAL trial [133, 134]. Co-inhibition of FLT3 and BCL2 was found to have synergistic effects [135]. A phase 1 study to test the safety and efficacy of adding venetoclax to gilteritinib in this patient population was conducted, and the result was reported [136]. A brief dose escalation of gilteritinib was followed by an expansion cohort with a final dose of 120 mg daily gilteritinib. Venetoclax 400 mg daily was used in combination with gilteritinib. Total of 43 patients were enrolled, among which 28 patients (65%) had previous history of Flt3 TKI exposure. Among 41 patients who are evaluable for response, 35 (85%) achieved CRc. Impressively high CRc rate (82%) was persistent in patients with prior Flt3 TKI exposure. These data compare favorably to the 52% CRc rate with single-agent gilteritinib in the ADMIRAL study. OS data await longer follow-up, but appear promising in preliminary analyses. Addition of venetoclax caused more cytopenia, which was manageable with shortened course of venetoclax on subsequent cycles upon CRc achieved. Therefore, gilteritinib in combination with venetoclax could be an optimal treatment for *Flt3*-mutated rrAML.

#### Venetoclax and azacitidine in IDH1/2 mutant AML

Hypomethylating agents combined with venetoclax are FDA approved and have been widely applied in clinical practice as first-line treatment for AML patients who are unfit for intensive chemotherapy. A study reported by Dr. Daniel Pollyea further analyzed the effect of this regimen on IDH1/2 mutant AML [137]. Data were pooled from the completed phase 1b (NCT02203773) and VIALE-A (NCT02993523) studies, in which azacitidine and venetoclax were applied to chemo-ineligible untreated AML patients. The focus was placed on patients with IDH1/2 mutations (*n* = 107). A higher CR/CRi rate was observed in IDH1/2 AML patients receiving venetoclax + azacitidine compared with placebo + azacitidine (78.5% vs. 10.7%). A substantially longer median OS was also achieved (24.5 vs. 6.2 months).

#### Enasidenib for newly diagnosed AML

Under the BEAT AML master trial, patients with newly diagnosed IDH2 mutant AML (> 60 year old) received enasidenib up to 5 cycles before response evaluation. Treatment continued for patients who achieved CR/CRi. Azacitidine was added to enasidenib for non-responders. Among the 60 patients who received enasidenib monotherapy as first line, 28 (47%) achieved CR/CRi, and median OS was 24.4 months. For the 17 enasidenib non-responders who received azacitidine + enasidenib subsequently, seven (41%) were able to obtain CR/CRi, and median OS was 8.9 months [138]. Therefore, enasidenib monotherapy appears to be a promising treatment for IDH2 mutant AML [139]. However, with the appealing data of venetoclax + azacitidine in the same patient population, further studies comparing the two regimens are needed for a conclusion. In addition, whether triple therapy adds additional benefit remains to be determined [140].

#### New agents under development

Several new agents showed promising results in treating AML. Magrolimab is a first-in-class mAb against CD47 that interferes with the CD47-SIRPα axis and inhibits the "don't eat me" signal used by cancer cells to avoid being ingested by macrophages [141, 142]. In a phase 1b study, Magrolimab combined with azacitidine was well tolerated and with decent efficacy in untreated AML patients who are unfit for intensive chemotherapy. Importantly, a promising response was observed in TP53-mutant AML with a 71% response rate, 48% CR rate and median OS of 12.9 months [143]. Targeting CD123 is also a promising direction. Flotetuzumab and vibecotamab are both CD123/CD3 BiTE. An update of phase 1/2 study of flotetuzumab in primary refractory AML reported a CR rate of 42% [144]. In a phase 1 study, vibecotamab achieved 14% ORR in heavily pretreated patients with rr AML [145]. Both BiTEs were well tolerated, CRS was a prominent toxicity, but it was generally manageable with pre-medications. Lintuzumab Ac225 is a radiolabeled anti-CD33 antibody. Preliminary results from a phase 1 study showed that combining lintuzumab with CLAG-M results in a promising CR/CRi (10/15, 67%) in relapsed/refractory AML [146].

### Update of ALL therapy from ASH 2020 Annual Meeting

#### Chemotherapy-free regimens for Ph + acute lymphoblastic leukemia (ALL)

GIMEMA LAL2116 study, a phase 2 single-arm trial, evaluated the first-line chemo-free combination of dasatinib and blinatumomab (Blin) for the induction and consolidation of newly diagnosed Ph + ALL [147]. Of the 63 patients enrolled, CR was reported to be 98%. Complete molecular response (CMR) was reported to be 60% after two cycles of Blin. A total of 24 patients went on to receive allogeneic HSCT. At a median follow-up of 18 months, OS was 95%. Therefore, this chemo-free, targeted and immunotherapeutic regimen led to high molecular response with few severe AEs in adult Ph + ALL.

A combination of ponatinib, venetoclax and dexamethasone was studied in a phase I/II trial for patients with R/R Ph + ALL [148] (Table 12). The starting dose of ponatinib was 45 mg daily, venetoclax daily (400 mg in dose level 1; up to 800 mg in dose level 2) and dexamethasone 40 mg IV/PO daily × 4 days each 28-day cycle. The primary endpoint was MTD of venetoclax in the combination regimen, and the phase II endpoint was CR/CRi rate. In the update from the ASH 2020 meeting, 6 pts were evaluable for safety and efficacy with 3 pts at venetoclax 400 mg and 3 pts at 800 mg daily, respectively. DLT has not been observed, and the MTD has not been reached. The 3 patients at 800 mg dose level all achieved CR. In conclusion, the chemo-free, 3-drug combination seems to be safe. The triplet oral agents showed high efficacy with venetoclax 800 mg daily in this heavily pretreated R/R Ph + ALL patients.

**Table 12:** Selected studies for ALL therapy from 2020 ASH annual meeting

Abstract #	Authors (reference)	Study agents	Phase	NCT No
465	Short et al. [148]	Ponatinib Venetoclax dexamethasone	I/II	03576547
3347	Ghobadi et al. [151]	AlloHSCT versus chemotherapy	Multicenter Retrospective	
1014	Short et al. [155]	MiniHCVD Inotuzumab ozogamicin Blinatumomab	II	
163	Jain et al. [158]	UCART22	I	04150497
499	Hu et al. [159]	CTA101	I	04154709

ALL, acute lymphoblastic leukemia; AlloHSCT, allogeneic hematopoietic stem cell transplantation; HCVD, fractionated cyclophosphamide, vincristine, dexamethasone

#### Allogeneic hematopoietic stem cell transplantation (AlloHSCT) for Ph + ALL in CR1: yes or no

Due to the high risk of relapse for Ph + ALL patients, traditionally alloHSCT has been the standard of care for adult patients in CR1 [149, 150]. The routine addition of TKI to the treatment regimens have led to significantly better CMR and outcomes. It becomes a common question now whether alloHSCT should be routinely recommended for these patients in CR1. A retrospective analysis of 186 adult patients who received TKI + induction therapy for Ph + ALL was reported at the ASH 2020 meeting [151]. In this report, 120 patients did not receive AlloHSCT in CR1 (chemo group), whereas 66 patients underwent alloHSCT as consolidation (HCT group). The median follow-up for survivors was 73.2 months (4.3–206 months range). There were no significant differences in OS and RFS between the two treatment groups (Table 12). More patients appeared to stay on TKIs as maintenance in the chemo group (92.5%) than those in the HCT group (47%). Overall, this retrospective analysis from 3 US centers appears to suggest that Ph + ALL with CMR in CR1 have similar long-term outcomes due to the possibility that higher transplant-related toxicities offset the lower relapse rate in the transplant group. This intriguing conclusion needs confirmation from a prospective randomized study.

#### MiniHCVD-INO-blinatumomab regimen: new protocol modification for patients ≥ 70

Both inotuzumab ozogamicin (INO) and blinatumomab (Blin) have been shown in randomized studies to be better than salvage chemotherapies for R/R ALL [3, 152]. MiniHCVD as a reduced-intensity induction regimen for older adults with ALL is being evaluated in combination with INO and Blin [4, 153, 154]. In a updated report, 73 patients have been enrolled, with 70 evaluable for efficacy [155]. The median follow-up was 45 months (range 2–98 months). The ORR was 98%. MRD negativity by flow cytometry was 96% overall. In total, 9% (6 pts) developed veno-occlusive disease (VOD). The 4-year CR and OS rates were 78% and 50%, respectively. There was higher death rate in remission in pts ≥ 70 years (45%) vs. 20% in pts 60–69 years of age (*p* = 0.03). Infection (*n* = 7) or development of MDS/AML (*n* = 3) were the main causes of death in remission. In conclusion, the miniHCVD-INO-Blin regimen is safe and effective for frontline therapy of pts age 60–69 years of age with Ph-negative ALL. Due to increased death rate in remission, miniHCVD chemotherapy was eliminated from the protocol for pts ≥ 70 years of age in this ongoing clinical trial.

#### Universal “off-the-shelf” CAR-T cells targeting CD22 for ALL therapy: UCART22 and CTA101

Four CD19-targeted CAR-T cell products have been approved for therapy of refractory B cell malignancies [5, 9, 28, 156]. CAR-T cells targeting new antigens are under active clinical development [6, 15]. Universal CAR-T cells have been in clinical trials [157]. UCART22 is a universal CAR-T cell product targeting CD22 with T cells derived from non-HLA-matched healthy donors. In an update at the ASH 2020 on the BALLI-01 study (NCT04150497), a phase I open-label dose-finding study, 5 patients have been treated, with 3 patients dosed at level 1 and 2 dosed at level 2 [158]. None of the 5 patients had serious TEAE, ICANS or DLT. In particular, there was no GVHD reported. Two patients achieved CRi. CD52 was knocked out in the UCART22 cells; therefore, alemtuzumab is being added to the lymphodepletion regimen in future cohorts with the goal to enhance the expansion and persistence of UCART22 after deeper depletion of patients’ T cells.

CTA101, a bi-cistronic, bispecific universal CAR-T cell agent targeting both CD19 and CD22, was also studied in a first-in-human phase I clinical trial [159]. Six patients have been enrolled, and 5 patients achieved CR/CRi with MRD negativity. All six patients developed CRS but all recovered. Remarkably, no GVHD was observed in this early report with short follow-up.

## Conclusions and perspectives

Four CD20-CD3 BiTEs for rrNHL in early-phase clinical trials were reported to have ORR and CR rates in the range of 50–67% and 20–33%, respectively, for patients with heavily treated rrDLCBL, including those with prior treatment failure of CD19 CAR-T cell therapy. Their long-term efficacy and safety profiles remain to be seen from larger clinical trials. Four CD19-targeted CAR-T cell products have been approved by US FDA for high-risk B cell malignancies, including axicabtagene ciloleucel, *tisagenlecleucel*, brexucabtagene autoleucel, and lisocabtagene maraleucel. Dual antigen-targeting CD19/20 and CD19/22 CAR-T cells aiming to reduce antigen escape appear promising in the early phase clinical trials. Further studies are needed to better understand the advantages, efficacy, long-term complications and major differences among these CAR-T cells. Ibrutinib, acalabrutinib, zanubrutinib and orelabrutinib covalently/irreversibly bind to the ATP binding site in the catalytic domain of BTK. They share the same mechanism of action, but differ in the off-target toxicities. LOXO-305, a non-covalent binding BTK inhibitor, is a very promising agent for patients with rrMCL including those after failing covalent BTKs.

The highlights of ASH 2020 in therapeutic advances on multiple myeloma (MM) credit to BCMA-targeted immunotherapy, led by BITEs, CAR-T/NK cells and ADCs. Belantamab mafodotin-blmf is the first FDA-approved ADC targeting BCMA. With multiple BCMA-targeted agents moving toward clinical applications, it is crucial to investigate the impacts of various sequential or combination therapeutic algorithms in the future studies.

Two JAK targeted inhibitors, ruxolitinib and fedratinib, have been approved for clinical therapy of myelofibrosis. Though ruxolitinib changed the landscape of MPN management and outlook, there are unmet needs to develop novel drugs or more effective therapeutic strategies for MPN patients. Novel JAK pathway inhibitors are under investigation as well [160, 161]. Combination of ruxolitinib with azacitidine and with pelabresib (CPI-0610, a BET inhibitor) is being studied for advanced MPNs [162]. Interestingly, interferon-alpha regained attention on its unique long-term effectiveness on certain MPN patients.

For CML, asciminib represents an allosteric TKI being effective against T315I mutation. It has shown advantage over bosutinib in the ASEMBL trial in highly refractory CML patients. Additional TKIs are shown to be active against T315I mutation and hold promise for clinical applications.

The combination of BTK inhibitor and BCL-2 inhibitor is highly efficacious in CLL. LOXO-305 appears to be promising for patients with highly refractory CLL. CD19-targeted CAR-T cells in combination with ibrutinib appeared to be efficacious for CLL refractory to both BTK and BCL-2 inhibitors. MRD-based endpoint is becoming a surrogate marker for PFS in CLL clinical trials.

With multiple targeted agents approved in recent years for AML therapy, combination regimens for newly diagnosed as well as R/R AML are under active clinical trials [127]. Magrolimab as a first-in-class CD47 mAb blocking the "do-not-eat-me" signal appears to be promising in early clinical trials.

Chemo-free regimen combining blinatumomab and TKI becomes a frontline option for Ph + ALL. MiniHCVD-INO-blinatumomab regimen has been in active clinical trials for elderly ALL patients. Further modification is being done to reduce toxic death in patients =  > 70. Universal CAR-T cells targeting CD22 or dual-targeting CD19/CD22 hold promise in early clinical trials with no GVHD.

## Data Availability

The material supporting the conclusion of this review has been included within the article.

## Abbreviations

ALL: Acute lymphoblastic leukemia
ADC: Antibody–drug conjugate
AlloSCT: Allogenic stem cell transplantation
BCMA: B-cell maturation antigen
BiTE: Bi-specific T cell engager
CAR: Chimeric antigen receptor
CR: Complete response
CRS: Cytokine release syndrome
DLT: Dose-limiting toxicity
DOF: Duration of follow-up
IMiD: Immunomodulatory drug
mAb: Monoclonal antibodies
DOR: Median duration of response
MM: Multiple myeloma
PFS: Median progression-free survival
MRD: Minimal/measurable residual disease
MTD: Maximal tolerated dose
NTX: Neurotoxicity
ORR: Overall response rate
OS: Overall survival
PD: Progressive disease
PI: Proteasome inhibitor
PR: Partial response
R/R (rr): Relapsed and/or refractory
SAE: Severe adverse event
sCR: Stringent complete response
TLS: Tumor lysis syndrome
TEAE: Treatment-emerging adverse event
TRD: Treatment-related death
VGPR: Very good partial response
